# Comparative Metabolic Profiling of Green and Purple Pakchoi (*Brassica Rapa* Subsp. *Chinensis*)

**DOI:** 10.3390/molecules23071613

**Published:** 2018-07-02

**Authors:** Jin Jeon, Chan Ju Lim, Jae Kwang Kim, Sang Un Park

**Affiliations:** 1 Department of Crop Science, Chungnam National University, 99, Daehak-Ro, Yuseong-gu, Daejeon 34134, Korea; jeonjin519@cnu.ac.kr; 2 Asia Seed Co., Ltd., 109-35, 518 Beon-gil, Gyeongchungdae-ro, Janghowon-eup, Icheon-si 17414, Korea; cabbage@asiaseed.co.kr; 3 Division of Life Sciences and Bio-Resource and Environmental Center, Incheon National University, Yeonsu-gu, Incheon 22012, Korea; kjkpj@inu.ac.kr

**Keywords:** pakchoi, phenylpropanoid, anthocyanin, glucosinolate, metabolic profiling

## Abstract

Pakchoi (*Brassica rapa* subsp. *chinensis*) is cultivated for its nutritional value, particularly with regard to vitamins, minerals and dietary fibers. However, limited metabolic information is available on the phyto-nutritional traits of pakchoi. Our GC-TOF MS analysis showed that green pakchoi has higher contents of carbon metabolism-associated metabolites such as sugars, sugar derivatives and inositol, while purple pakchoi has higher levels of nitrogen metabolism-associated metabolites such as amino acids and amino acid derivatives. To compare the content and composition of secondary metabolites in green and purple pakchoi, we analyzed phenylpropanoid-derived compounds and anthocyanins in mature leaves using an HPLC-UV system. This analysis identified 9 phenylpropanoid-derived compounds and 12 anthocyanins in the mature leaves of green and purple pakchoi. The level of rutin was significantly higher in purple pakchoi compared with green pakchoi, consistent with the expression of phenylpropanoid biosynthetic genes in the two pakchoi cultivars. The data obtained from this comprehensive metabolic profiling would be helpful to improve our understanding of the nutritional values of pakchoi cultivars as food sources.

## 1. Introduction

The *Brassica* genus is the largest and most diverse within the Brassicaceae family and includes many economically important crop plants that are cultivated worldwide. This genus includes vegetables such as Chinese cabbage, turnip, cabbage, kale, broccoli, cauliflower, kohlrabi and Brussels sprouts that are known to be rich in phytochemicals and soluble fibers [1,2,3]. In addition, several cultivars of rapeseed are used in the production of canola oil and the seeds of some *Brassica* species are used in the production of the condiment mustard.

Pakchoi (*Brassica rapa* subsp. *chinensis*) is an important crop that is widely cultivated and consumed in Asia and the level of consumption is also rising in countries in Northern Europe and elsewhere. The young leaves of pakchoi are consumed as part of salads or a garnish, the mature plants can be eaten steamed or briefly cooked. The plant is known by a variety of common names, such as “bok choy, pak choy and buk choy”. Pakchoi contains numerous health-benefiting compounds, such as glucosinolates, flavonoids, vitamins and minerals [4,5]. Kaempferol, isorhamnetin, quercetin and hydroxycinnamic acid derivatives have also been detected in various cultivars of pakchoi [6,7]. In the purple pakchoi cultivar ‘Zi He’, 15 types of anthocyanin have been identified by analyses using high-performance liquid chromatography-electrospray ionization tandem mass spectrometry (HPLC-ESI-MS/MS) [8]. However, the metabolome of pakchoi still remains poorly understood.

Plants produce numerous metabolites that play critical roles in growth, development and reproduction. In addition to primary metabolites, various secondary metabolites are produced in response to external stimuli or endogenous signals. Secondary metabolites play important roles for plant defenses against herbivores and pathogens [9]. Most secondary metabolites are created by modification of primary metabolites or substrates of primary metabolites. Therefore, secondary metabolite biosynthesis is related to their primary metabolite origin. Phenylpropanoids are a class of secondary metabolites synthesized from the amino acid phenylalanine that is a product of the shikimate pathway [10,11,12]. The phenylpropanoid pathway generates various metabolites, including lignin, chlorogenic acid, epicatechin, rutin, quercetin, kaempferol and anthocyanin (Figure S1). Rutin (quercetin-3-rhamnosyl glucoside) is a low molecular weight polyphenolic compound that is found in many plants, including *Fagopyrum tataricum* [13], *Morus alba* [14] and *Lycium chinense* [15]. Recent study has reported that rutin contains various pharmacological functions and can act as an antioxidant [16], cytoprotective [17], vasoprotective [18], antiproliferative [19], antithrombotic [20] and cardioprotective agent [21]. In addition to rutin, quercetin and kaempferol show high antioxidant activities [22]. Anthocyanins are a kind of flavonoids and are responsible for producing different colors in plants and have been exploited for applications in the food industry [23,24,25].

In this study, we used an HPLC system to compare the content and composition of phenylpropanoids derived compounds and anthocyanins in mature leaves of green and purple pakchoi cultivars. We also performed metabolic profiling analysis to compare the differences in metabolites between green and purple pakchoi using a GC-TOF MS system. The results of these study will enhance our understanding of metabolic information in pakchoi cultivars.

## 2. Results and Discussion

### 2.1. Analysis of Polar Metabolites in Green and Purple Pakchoi 

**Figure 1:**
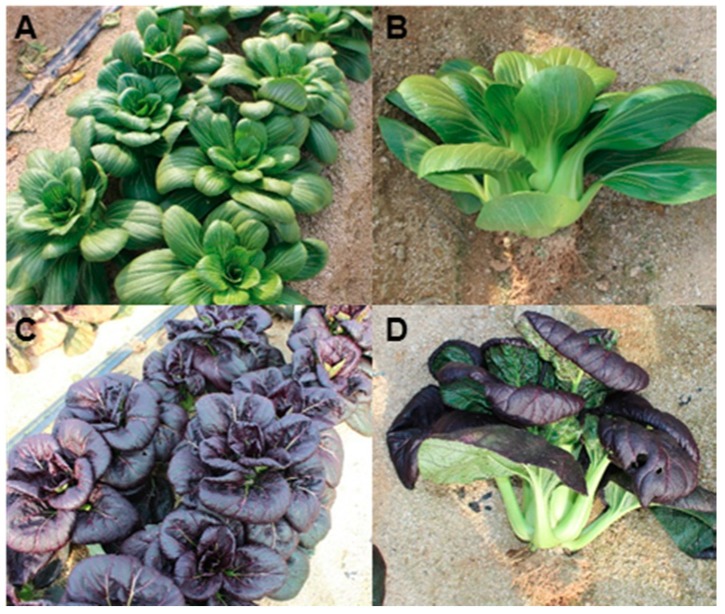
Photographs of mature pakchoi. Green pakchoi cultivar ‘8210’ (**A**,**B**) and purple pakchoi cultivar ‘8389’ (**C**,**D**).

Pakchoi is a small plant that has an upright form and does not form a head. Mature pakchoi reaches a height of 30–45 cm (Figure 1). Pakchoi cultivar ‘8210’ has smooth and dark green leaf blades (Figure 1A,B), while cultivar ‘8389’ had curled and purple leaf blade (Figure 1C,D).

**Figure 2:**
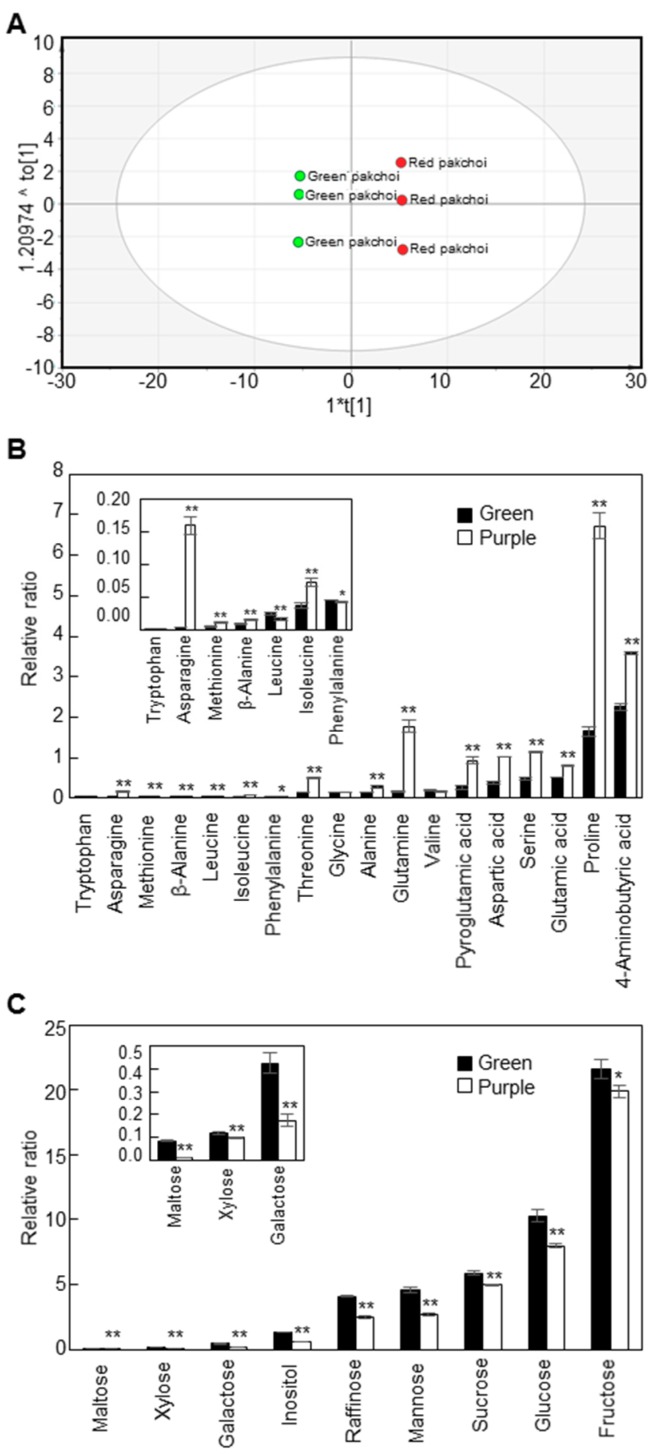
Metabolic profiling of two pakchoi cultivars. The OPLS-DA score plot (**A**), contents of amino acids and amino acid derivatives (**B**) and contents of sugars and sugar derivatives (**C**) in green and purple pakchoi. Contents of metabolites were measured in 2-month-old green and purple pakchoi (μg g^−1^ dry weight). The quantitative calculations of all metabolites were based on the peak area ratios relative to that of the ribitol as an internal standard (IS). The peak area of metabolite was normalized to peak area of the IS, which the peak area ratio was used for the relative quantification. Each value represents the mean of three technical replicates and error bars are SDs. Asterisks indicate significant differences the purple pakchoi compared with the green pakchoi using Student’s *t* test (* *p* < 0.05; ** *p* < 0.01).

We compared the content and composition of polar metabolites in mature leaves of green and purple pakchoi using GC-TOF MS analysis (Figure 2). A total of 43 metabolites, including amino acids, sugars and organic acids, were detected in mature green and purple pakchoi leaves. The corresponding retention times, fragment patterns and their ion masses are given in Table S1. The quantitative calculations of all metabolites were based on the peak area ratios relative to that of ribitol as an internal standard (IS). We performed OPLS-DA to visualize the structure of the multi-dimensional data and found that green and purple pakchoi could be separated by the components (Figure 2A). The quality of the OPLS-DA models can be explained by the goodness of fit (R^2^) and predictive ability (Q^2^). The R^2^ and Q^2^ values were 0.802 and 0.999, respectively. The model “if Q^2^ > 0.8” is usually considered to have an excellent predictive ability. The corresponding loading plot shows the metabolites responsible for separation on the score plot (Figure S2A). All sugars were clustered on the negative side of the loading plot, indicating that the levels of the sugars in purple pakchoi were lower than those in green pakchoi. The values of variables important in the projection (VIP) explain how the contribution of the variables to the projection and a VIP value of >1 is used as the criterion to identify important variables to the model [26]. Among the 43 metabolites, 31 metabolites had VIP values >1 (Figure S2B). These findings suggest that primary metabolism differed significantly between green and purple pakchoi.

As shown in the loading plot data, most of the amino acids and amino acid derivatives were higher in purple pakchoi than in green pakchoi, excepting leucine and phenylalanine (Figure 2B). Among 18 amino acids and amino acid derivatives, 15 metabolites showed significant differences in their means between green pakchoi and purple pakchoi. Especially, asparagine content increased by 32.84-fold in purple pakchoi compared with green pakchoi. In mice, asparagine is required for development of the brain and is needed to maintain balance in the central nervous system [27]. The sugar and sugar derivative contents were significantly lower in purple pakchoi compared with green pakchoi (Figure 2C). Maltose, xylose, galactose, inositol, raffinose, mannose, sucrose, glucose and fructose contents were 6.27-, 1.19-, 2.42-, 2.26-, 1.64-, 1.71-, 1.19-, 1.29- and 1.09-fold lower in purple pakchoi than in green pakchoi, respectively. Our GC-TOF MS analysis revealed that nicotinic acid, lactic acid, quinic acid, shikimic acid, glyceric acid, fumaric acid, succinic acid, citric acid and malic acid were present in mature leaves of green and purple pakchoi (Table S2). Nicotinic acid, also known as vitamin B_3_ and niacin, was 2.52-fold higher in purple pakchoi than in green pakchoi. Lactic acid also increased by 1.74-fold in purple pakchoi compared with green pakchoi. Quinic acid, a component of cyclic polyol, increased by 3.71-fold in purple pakchoi compared with green pakchoi. Glyceric acid, a natural three-carbon sugar acid derived from glycerol, was 7.37-fold lower in purple pakchoi compared with green pakchoi. Some organic acids derived from the tricarboxylic acid (TCA) cycle, such as citric acid, fumaric acid and malic acid, showed few significant changes between green pakchoi and purple pakchoi. Citric acid was increased 1.35-fold, whereas fumaric acid and maleic acid were 1.57- and 1.30-fold lower in purple pakchoi compared with green pakchoi, respectively. The level of malic acid was considerably higher than any other organic acids. The content of malic acid in the green pakchoi was 45.0, 29.5 and 5.9 times higher than that in the fumaric acid, succinic acid and citric acid, respectively. In the red pakchoi, the content of malic acid was 54.2, 22.7 and 3.4 times higher than that in the fumaric acid, succinic acid and citric acid, respectively. The high content of malic acid may play an important role in energy source and carbon skeletons for the formation of amino acids in cell. Succinic acid did not show any significant differences between green pakchoi and purple pakchoi.

### 2.2. Identification of Phenylpropanoid Biosynthetic Genes in Pakchoi Cultivars

**Figure 3:**
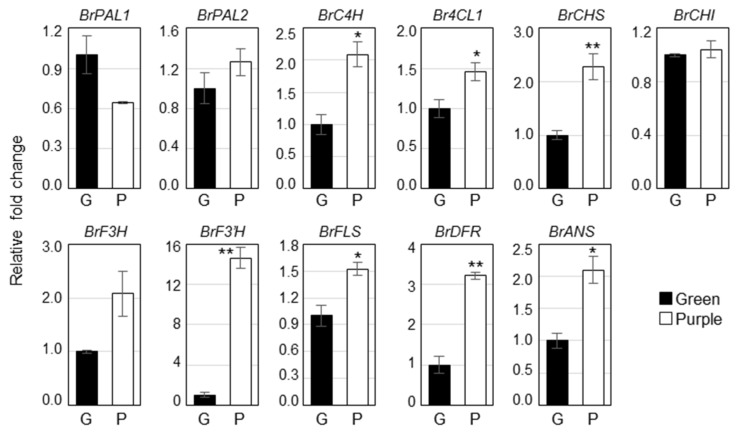
Expression of the phenylpropanoid biosynthetic genes in green and purple pakchoi. Total RNA isolated from 2-month-old green and purple pakchoi were subjected to quantitative real time RT-PCR. Relative fold changes were plotted after normalization to *EF1α*. The means ± S.E. from three biological and technical replicate experiments were plotted. Asterisks indicate significant differences compared with the green pakchoi using Student’s *t* test (* *p* < 0.05; ** *p* < 0.01). *PAL*, phenylalanine ammonia-lyase; *C4H*, cinnamate 4-hydroxylase; *4CL*, 4-coumarate-CoA ligase; *CHS*, chalcone synthase; *CHI*, chalcone isomerase; *F3H*, flavanone-3-hydroxylase; *F3*′*H*, flavonoid-3′-hydroxylase; *FLS*, flavonol synthase; *DFR*, dihydroflavonol reductase; *ANS*, anthocyanin synthase.

To identify the molecular mechanisms of different pigment accumulation in two pakchoi cultivars, we analyzed the expression of phenylpropanoid biosynthetic genes such as *BrPAL1*, *BrPAL2*, *BrC4H*, *Br4CL1*, *BrCHS*, *BrCHI*, *BrF3H*, *BrF3*′*H*, *BrFLS*, *BrDFR* and *BrANS* in green and purple pakchoi (Figure 3). Most of the phenylpropanoid biosynthetic genes were significantly upregulated in purple pakchoi compared to the green pakchoi. The expression of *BrC4H*, *BrCHS*, *BrCHI*, *BrF3*′*H*, *BrFLS*, *BrDFR* and *BrANS* was increased over 2-fold in purple pakchoi compared with green pakchoi. Particularly, the expression of *F3*′*H*, which is a key gene of the rutin biosynthesis, was significantly increased by over 14-fold in purple pakchoi, predicting that the content of rutin might more highly increase in purple pakchoi. On the other hand, the expression of *BrPAL1*, *BrPAL2*, *BrCHI* and *BrF3H* was not statistically difference between the two pakchoi cultivar.

### 2.3. Analysis of Phenylpropanoid Contents in Green and Purple Pakchoi 

We compared the content and composition of phenylpropanoid-derived compounds in mature leaves of green and purple pakchoi using an HPLC-UV system. Our analysis showed that seven phenylpropanoid-derived compounds, namely, chlorogenic acid, caffeic acid, epicatechin, *p*-coumaric acid, ferulic acid, rutin and *trans*-cinnamic acid, were present in mature leaves of green pakchoi (Table 1). Besides these compounds, purple pakchoi had two additional compounds, 3.78 μg/g quercetin and 4.03 μg/g kaempferol, respectively. Among the nine detected phenylpropanoid-derived compounds, the levels of caffeic acid and *p*-coumaric acid were 2.08- and 1.95-fold higher in green pakchoi than in purple pakchoi, respectively. The levels of the other seven compounds were higher in purple pakchoi than in green pakchoi. The amount of rutin in purple pakchoi compared to green pakchoi was much higher than those of the other compounds. The rutin was present 393.43 μg/g dry weight in purple pakchoi and this was 57.1-fold higher than in green pakchoi. The total content of phenylpropanoids was 3.33-fold higher in purple pakchoi than in green pakchoi. This analysis showed that purple pakchoi had higher levels of rutin, quercetin and kaempferol than green pakchoi, indicating that purple pakchoi may have higher antioxidant activities than green pakchoi.

### 2.4. Analysis of Anthocyanin Contents in Green and Purple Pakchoi 

To determine whether the pigmentation of purple pakchoi is caused by anthocyanins, we analyzed soluble anthocyanins in mature leaves of green and purple pakchoi using an HPLC-Q-Trap MS system. Anthocyanins were not detected in green pakchoi, whereas 12 anthocyanins were separated and characterized from purple pakchoi (Table 2, Figure S3). The major anthocyanins were cyanidin and cyanidin derivatives in purple pakchoi but not delphinidin or pelargonidin. The major acylated anthocyanins were cyanidin 3-diglucoside-5-glucoside derivatives with several acylated groups such as *p*-coumaric acid, caffeic acid, ferulic acid and sinapinic acid. The total anthocyanin content of purple pakchoi was 1.91 mg/g dry weight. Among the 12 anthocyanins, cyanidin 3-(sinapoyl)diglucoside-5-glucoside was the most abundant (0.35 mg/g dry weight), followed by cyanidin 3-(sinapoyl)(sinapoyl)diglucoside-5-glucoside (0.34 mg/g dry weight). The composition of anthocyanins found in purple pakchoi was similar to that reported by Zhang et al. (2014). In their study, Zhang and coworkers showed that 15 anthocyanins were present in ‘Zi He’, a purple cultivar and that no anthocyanins were detected in the mature leaves of ‘Su Zhouqing’, a green cultivar. The principal anthocyanins identified in ‘Zi He’ were cyanidin and cyanidin derivatives but not petunidin 3,5-diglucoside.

## 3. Materials and Methods 

### 3.1. Plant Material and Growth Conditions 

The green pakchoi cultivar ‘8210’ and purple pakchoi cultivar ‘8389’ were grown into seedbeds containing soil to 3 weeks after germination. The seedlings were grown under field conditions at Asia Seeds Ltd., Korea with mean minimum temperature 15 °C and mean maximum temperature 27 °C. Mature plants at approximately two months after sowing were separated from root tissue. Harvested samples were frozen in liquid nitrogen and stored at −70 °C until it could be used for QRT-PCR and HPLC-UV analysis.

### 3.2. Polar Metabolite Profiling by GC-TOF MS 

Polar metabolites were extracted and analyzed as described previously [28,29]. The powdered samples (10 mg) were dissolved in 1 mL of 2.5:1:1 (*v/v/v*) methanol:water:chloroform and performed mixing at 1200 rpm for 30 min at 37 °C. The polar phase was transferred into a new tube after centrifugation and 0.4 mL of water was added. The mixed samples were centrifuged at 16,000× *g* for 5 min and then the methanol/water phase was freeze-dried for 16 h. The samples were redissolved in 80 μL of methoxyamine hydrochloride (20 mg/mL) in pyridine and incubated for 90 min at 30 °C. This samples were derivatized with 80 μL of MSTFA at 37 °C for 30 min. Sixty microliter ribitol (0.2 mg/mL) was used as an internal standard (IS). After derivatization, 1 µL samples were analyzed into an Agilent 7890A gas chromatograph (Agilent, Atlanta, GA, USA) connected to a Pegasus HT TOF mass spectrometer (LECO, St. Joseph, MI, USA) with a split ratio of 1:25. The column was 30 m × 0.25 mm ID fused-silica capillary column coated with 0.25-µm CP-SIL 8 CB low bleed (Varian Inc., Palo Alto, CA, USA). The injection temperature was set to 230 °C. The helium gas (1.0 mL/min) was used as carrier gas. The temperature program was as follows: 2 min isothermal heating at 80 °C, followed by an increase to 320 °C at 15 °C/min and a 10 min hold at 320 °C. The transfer line and ion-source temperatures were set at 250 °C and 200 °C, respectively. Mass scanning range was 85–600 *m/z* and the detector voltage was set at 1700 V.

Component detection and automated deconvolution were conducted with a ChromaTOF software. The compounds identified in libraries derived from the National Institute of Standards and Technology (NIST) and the in-house libraries for standard chemicals.

### 3.3. RNA Extraction and QRT-PCR 

The pakchoi cultivar were immediately frozen in liquid nitrogen following treatment. Total RNA was isolated from frozen pakchoi cultivars using total RNA mini kit (Geneaid Biotech Ltd., New Taipei City, Taiwan). QRT-PCR analysis was conducted using a QuantiTect SYBR Green RT-PCR kit (Qiagen, Valencia, CA, USA) in a CFX96TM real-time PCR detection system (Bio-Rad, Hercules, CA, USA). Data analysis and determination of reaction specificities were performed as described previously [30]. Gene expression was normalized to that of the *BrEF1α* gene, used as a housekeeping gene. All QRT-PCR assays were conducted as the mean of three different biological experiments and subjected to statistical analysis. QRT-PCR product sizes and primer sequences are provided in Table S3.

### 3.4. Extraction and HPLC Analysis of Phenylpropanoids 

Phenylpropanoids were extracted from 100 mg dried samples with 3 mL 80% methanol at 25 °C for 1 h. The extracts were filtered through a 0.45 μm PTFE syringe filter (Advantec DISMIC-13HP, Toyo Roshi Kaisha, Ltd., Tokyo, Japan) after centrifugation. Phenylpropanoids were measured on a NS-4000 HPLC apparatus (Futecs, Daejeon, Korea) with a C_18_ column (250 × 4.6 mm, 5 μm; RStech, Daejeon, Korea) and monitored at 280 nm. A 20 µL injection was separated using a mixture of 0.15% acetic acid (solvent A) and 100% methanol (solvent B) at a flow rate of 1.0 mL/min. The elution condition was set as: 5% solvent B, followed by a linear gradient from 0 to 80% solvent B over 93 min and then a hold at 5% solvent B for an additional 5 min. Chlorogenic acid, caffeic acid, epicatechin, *p*-coumaric acid, ferulic acid, rutin, *trans*-cinnamic acid, quercetin and kaempferol were purchased from Sigma-Aldrich (St. Louis, MO, USA). Different compounds were quantified on the basis of peak areas and the concentrations were calculated as equivalents of representative standard compounds. The linear equations and regression coefficients for chlorogenic acid, caffeic acid, epicatechin, *p*-coumaric acid, ferulic acid, rutin, *trans*-cinnamic acid, quercetin and kaempferol were *y* = 52135.7*x* − 57351.96 with *r*^2^ = 0.999, *y* = 101427.93*x* − 19999.04 with *r*^2^ = 0.999, *y* = 16656.06*x* − 8837.11 with *r*^2^ = 0.998, *y* = 172376.91*x* − 2288.50 with *r*^2^ = 0.998, *y* = 115029.24*x* − 16713.48 with *r*^2^ = 0.998, *y* = 25662.41*x* – 3352.89 with *r*^2^ = 0.999, *y* = 323515.13*x* – 3738.31 with *r*^2^ = 0.998, *y* =40520.94*x* + 12547.81 with *r*^2^ = 0.997 and *y* = 68166.24*x* – 4265.15 with *r*^2^ = 0.998, respectively. Means ± SD were determined from three replicates.

### 3.5. Extraction and HPLC Analysis of Anthocyanins

For anthocyanin analysis, 100 mg of dried powder was extracted with 2 mL water:formic acid (95:5 *v/v*) in a sonicator for 20 min and filtered in a brown vial. Anthocyanins were measured by an Agilent 1200 series HPLC (Santa Clara, CA, USA) connected to a 4000 Q-Trap LC-ESI-MS/MS system (Applied Biosystems, Foster City, CA, USA) with a 4 μm POLAR-RP 80A column (Phenomenex, Torrance, CA, USA), as described previously [30]. Each reactant was detected at a wavelength of 520 nm. A mixture of solvent A (water, 0.5% formic acid) and solvent B (acetonitrile, 0.5% formic acid) was used in mobile phase. The gradient program was set as: 0–8 min, 5–10% solvent B; 8–13 min, 10–13% solvent B; 13–15 min, 13% solvent B; 15–18 min, 13–15% solvent B; 18–25 min, 15% solvent B; 25–30 min, 15–18% solvent B; 30–35 min, 18% solvent B; 35–40 min, 18–21% solvent B; 40–45 min, 21% solvent B; and 45.1–50 min, 5% solvent B. The interpretation of the mass spectra was carried out by comparison with our database and published database [31,32]. The anthocyanin content was calculated by comparing the HPLC peak area with that of cyanidin-3-*O*-glucoside (Fujicco Co., Ltd., Kobe, Japan) as an external standard. Means ± SD were determined from three replicates.

### 3.6. Statistical Analysis 

The relative data acquired from polar metabolites profiling were subjected to orthogonal projections to latent structures discriminant analysis (OPLS-DA; SIMCA-P version 13.0; Umetrics, Umeå, Sweden) to inspect which components separated between green and purple pakchoi samples [33]. The OPLS-DA output consisted of score plots for visualizing the contrast between different samples and loading plots to explain the cluster separation. The data file was scaled with unit variance scaling before all the variables were subjected to OPLS-DA. Statistical analysis was performed with SPSS statistics 22 software (IBM Corp., Armonk, NY, USA) using Student’s *t* test. All data are given as the mean values and standard deviation of triplicate experiments.

## 4. Conclusions

**Figure 4:**
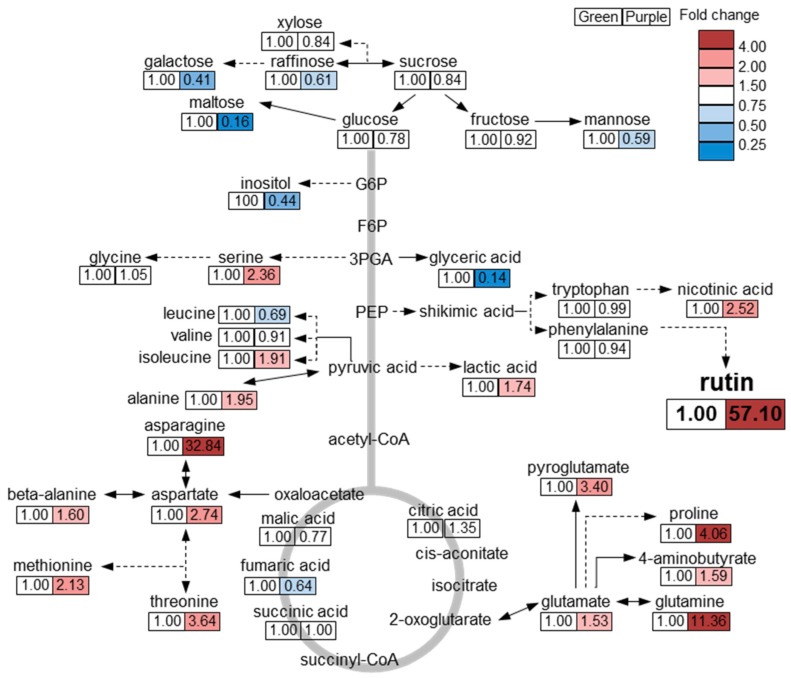
Overview of metabolites differing between green and purple pakchoi. The response ratio of purple to green pakchoi for each metabolite is shown. Red and blue colors indicate ratios of more than 1 and less than 1, respectively.

In conclusion, we obtained a comprehensive metabolic profile, including secondary metabolites, of green and purple pakchoi cultivars. Overall, the content of phenylpropanoids and anthocyanins was higher in purple pakchoi than in green pakchoi. In particular, the amount of rutin was considerably higher in purple pakchoi than in green pakchoi. Quercetin, kaempferol and anthocyanins were only detected in purple pakchoi. These phenylpropanoid compounds and their precursors act as antioxidants to inhibit the synthesis of reactive oxygen species (ROS) in plants [16,22]. The increased content of these metabolites in purple pakchoi suggests that it may have a higher antioxidant activity than green pakchoi. Based on metabolic profiling by GC-TOF MS analysis, we draw the following overview regarding metabolic differences between green and purple pakchoi (Figure 4). The levels of representative carbon (C) metabolism-associated metabolites (e.g., sugar, sugar derivative and inositol) were higher in green pakchoi than in purple pakchoi. Carbon metabolism is closely related to plant growth and development. Among the sugars and sugar derivatives, the content of maltose was notably increased in green pakchoi compared with purple pakchoi. The levels of nitrogen (N) metabolism-associated metabolites (e.g., amino acid and amino acid derivative) were generally increased in purple pakchoi compared with green pakchoi. Particularly, an increase in amino acids from the TCA cycle (e.g., asparagine, methionine, threonine, glutamine, pyroglutamate, aspartate, serine and proline) was observed in purple pakchoi compared with green pakchoi. A comparison of the metabolic profiles of green and purple pakchoi suggests that green pakchoi has a higher content of C metabolism–associated metabolites, while purple pakchoi has a higher level of N metabolism–associated metabolites and other secondary metabolites. These comprehensive metabolic data provide useful information for future research on pakchoi bio-engineering and the possibility of using single or combinations of metabolites as markers.

## Figures and Tables

**Table 1:** Phenylpropanoid contents in green and purple pakchoi (μg g^−1^ dry weight).

	Green	Purple
Chlorogenic acid	12.00 ± 0.52	14.33 ± 0.28 *
Caffeic acid	68.15 ± 4.48	32.77 ± 3.58 **
Epicatechin	58.47 ± 5.46	76.93 ± 10.50 *
*p*-Coumaric acid	9.23 ± 2.07	4.74 ± 0.32 *
Ferulic acid	9.41 ± 0.04	17.83 ± 0.91 **
Rutin	6.89 ± 1.64	393.43 ± 12.28 **
*trans*-Cinnamic acid	0.45 ± 0.01	0.78 ± 0.25
Quercetin	0.00 ± 0.00	3.78 ± 1.35 **
Kaempferol	0.00 ± 0.00	4.03 ± 1.10 **
Total	164.58 ± 3.21	548.62 ± 17.95 **

Total phenylpropanoid contents were measured in 2-month-old green and purple pakchoi (μg g^−1^ dry weight). Each value represents the mean of three technical replicates and error bars are SDs. Asterisks indicate significant differences the purple pakchoi compared with the green pakchoi using Student’s *t* test (* *p* <0.05; ** *p* < 0.01).

**Table 2:** Anthocyanin contents in green and purple pakchoi (mg g^−1^ dry weight).

No.	Trivial Names	Green	Purple
1	Cyanidin 3-diglucoside-5-glucoside	ND	0.02 ± 0.00
2	Cyanidin 3-(sinapoyl)diglucoside-5-glucoside	ND	0.18 ± 0.00
3	Cyanidin 3-(caffeoyl)(*p*-coumaroyl)diglucoside-5-glucoside	ND	0.02 ± 0.00
4	Cyanidin 3-(glycopyranosyl-sinapoyl)diglucoside-5-glucoside	ND	0.11 ± 0.00
5	Unknown	ND	0.05 ± 0.00
6	Unknown	ND	0.08 ± 0.00
7	Cyanidin 3-(*p*-coumaroyl)(sinapoyl)triglucoside-5-glucoside	ND	0.03 ± 0.00
8	Unknown	ND	0.03 ± 0.00
9	Unknown	ND	0.04 ± 0.00
10	Unknown	ND	0.06 ± 0.00
11	Cyanidin 3-(sinapoyl)glucoside-5-glucoside	ND	0.06 ± 0.00
12	Cyanidin 3-(*p*-coumaroyl)diglucoside-5-glucoside	ND	0.08 ± 0.00
13	Unknown	ND	0.02 ± 0.00
14	Cyanidin 3-(sinapoyl)diglucoside-5-glucoside	ND	0.35 ± 0.01
15	Unknown	ND	0.05 ± 0.00
16	Cyanidin 3-(*p*-coumaroyl)(sinapoyl)diglucoside-5-glucoside	ND	0.03 ± 0.00
17	Cyanidin 3-(feruloyl)(sinapoyl)diglucoside-5-glucoside	ND	0.12 ± 0.01
18	Unknown	ND	0.05 ± 0.00
19	Cyanidin 3-(sinapoyl)(sinapoyl)diglucoside-5-glucoside	ND	0.34 ± 0.01
20	Cyanidin 3-(feruloyl)(sinapoyl)diglucoside-5-glucoside	ND	0.20 ± 0.00
Total		0.00 ± 0.00	1.91 ± 0.03

Total anthocyanin contents were measured in 2-month-old purple pakchoi (mg g^−1^ dry weight). Each value represents the mean of three technical replicates and error bars are SDs. ND, not detected.

## Supplementary Materials

The following are available online, Table S1: Chromatographic and spectrometric data of the metabolites identified by GC-TOFMS, Table S2: Contents of organic acids and other metabolites in green and purple pakchoi, Table S3: Primers used in this work, Figure S1: Schematic representation of the phenylpropanoid biosynthetic pathway in plants. PAL, phenylalanine ammonia-lyase; C4H, cinnamate 4-hydroxylase; 4CL, 4-coumarate-CoA ligase; CHS, chalcone synthase; CHI, chalcone isomerase; F3H, flavanone-3-hydroxylase; F3′H, flavonoid-3′-hydroxylase; FLS, flavonol synthase; DFR, dihydroflavonol reductase; ANS, anthocyanin synthase; 3GT, flavonoid 3-*O*-glucosyltransferase; RT, 3-*O*-rhamnosyltransferase; COMT, caffeic O-methyltransferase; HQT, hydroxycinnamoyl-CoA quinate hydroxycinnamoyltransferase; C3H, 4-coumarate 3-hyroxylase; and ANR, anthocyanidin reductase. Bold letters indicate the genes or compounds measured in this study, Figure S2: The loading plot (A) and influence variables used to create a discrimination model for green and purple pakchoi (B). Variable important in the projection (VIP) was identified from the OPLS-DA model, Figure S3: HPLC profiles of anthocyanins in the purple pakchoi (cultivar #8389). The peak numbers indicate the anthocyanins in Table 2.

## Abbreviations

HPLC-UV	high-performance liquid chromatography-ultraviolet
GC-TOF MS	gas chromatography-time of flight mass spectrometry;
HPLC-ESI-MS/MS	high-performance liquid chromatography-electrospray ionization tandem mass spectrometry
TMS	trimethylsilyl
PCA	principal component analysis
OPLS-DA	orthogonal projections to latent structures discriminant analysis

## References

[1] Cartea M.E., Francisco M., Soengas P., Velasco P. (2010). Phenolic Compounds in Brassica Vegetables. Molecules.

[2] Jahangir M., Kim H.K., Choi Y.H., Verpoorte R. (2009). Health-Affecting Compounds in Brassicaceae. Compr. Rev. Food Sci. Food Saf..

[3] Park W.T., Kim J.K., Park S., Lee S.W., Li X., Kim Y.B., Uddin M.R., Park N.I., Kim S.J., Park S.U. (2012). Metabolic profiling of glucosinolates, anthocyanins, carotenoids and other secondary metabolites in kohlrabi (*Brassica oleracea* var. gongylodes). J. Agric. Food Chem..

[4] Bhandari S.R., Jo J.S., Lee J.G. (2015). Comparison of Glucosinolate Profiles in Different Tissues of Nine Brassica Crops. Molecules.

[5] Harbaum B., Hubbermann E.M., Wolff C., Herges R., Zhu Z., Schwarz K. (2007). Identification of Flavonoids and Hydroxycinnamic Acids in Pak Choi Varieties (*Brassica campestris* L. ssp. chinensis var. communis) by HPLC–ESI-MS n and NMR and Their Quantification by HPLC–DAD. J. Agric. Food Chem..

[6] Harbaum B., Hubbermann E.M., Zhu Z., Schwarz K. (2008). Free and bound phenolic compounds in leaves of pak choi (*Brassica campestris* L. ssp. chinensis var. communis) and Chinese leaf mustard (*Brassica juncea* Coss). Food Chem..

[7] Rochfort S.J., Imsic M., Jones R., Trenerry V.C., Tomkins B. (2006). Characterization of Flavonol Conjugates in Immature Leaves of Pak Choi [*Brassica rapa* L. ssp. chinensis L. (Hanelt.)] by HPLC-DAD and LC-MS/MS. J. Agric. Food Chem..

[8] Zhang Y., Chen G., Dong T., Pan Y., Zhao Z., Tian S., Hu Z. (2014). Anthocyanin accumulation and transcriptional regulation of anthocyanin biosynthesis in purple bok choy (*Brassica rapa* var. chinensis). J. Agric. Food Chem..

[9] Wittstock U., Gershenzon J. (2002). Constitutive plant toxins and their role in defense against herbivores and pathogens. Curr. Opin. Plant Biol..

[10] Fraser C.M., Chapple C. (2011). The Phenylpropanoid Pathway in Arabidopsis. Arabidopsis Book.

[11] Herrmann K.M., Weaver L.M. (1999). The shikimate pathway. Annu. Rev. Plant Physiol. Plant Mol. Biol..

[12] Vogt T. (2010). Phenylpropanoid biosynthesis. Mol. Plant.

[13] Li X., Thwe A.A., Park N.I., Suzuki T., Kim S.J., Park S.U. (2012). Accumulation of phenylpropanoids and correlated gene expression during the development of tartary buckwheat sprouts. J. Agric. Food Chem..

[14] Zhao S., Park C.H., Li X., Kim Y.B., Yang J., Sung G.B., Park N.I., Kim S., Park S.U. (2015). Accumulation of Rutin and Betulinic Acid and Expression of Phenylpropanoid and Triterpenoid Biosynthetic Genes in Mulberry (*Morus alba* L.). J. Agric. Food Chem..

[15] Zhao S., Tuan P.A., Li X., Kim Y.B., Kim H., Park C.G., Yang J., Li C.H., Park S.U. (2013). Identification of phenylpropanoid biosynthetic genes and phenylpropanoid accumulation by transcriptome analysis of Lycium Chinense. BMC Genom..

[16] Boyle S.P., Dobson V.L., Duthie S.J., Hinselwood D.C., Kyle J.A., Collins A.R. (2000). Bioavailability and efficiency of rutin as an antioxidant: A human supplementation study. Eur. J. Clin. Nutr..

[17] Potapovich A.I., Kostyuk V.A. (2003). Comparative study of antioxidant properties and cytoprotective activity of flavonoids. Biochemistry.

[18] Tang D.Q., Wei Y.Q., Gao Y.Y., Yin X.X., Yang D.Z., Mou J., Jiang X.L. (2011). Protective effects of rutin on rat glomerular mesangial cells cultured in high glucose conditions. Phytother. Res..

[19] Santos B.L., Silva A.R., Pitanga B.P., Sousa C.S., Grangeiro M.S., Fragomeni B.O., Coelho P.L., Oliveira M.N., Menezes-Filho N.J., Costa M.F. (2011). Antiproliferative, proapoptotic and morphogenic effects of the flavonoid rutin on human glioblastoma cells. Food Chem..

[20] Sheu J.R., Hsiao G., Chou P.H., Shen M.Y., Chou D.S. (2004). Mechanisms involved in the antiplatelet activity of rutin, a glycoside of the flavonol quercetin, in human platelets. J. Agric. Food Chem..

[21] Ziaee A., Zamansoltani F., Nassiri-Asl M., Abbasi E. (2009). Effects of rutin on lipid profile in hypercholesterolaemic rats. Basic Clin. Pharmacol. Toxicol..

[22] Nijveldt R.J., van Nood E., van Hoorn D.E., Boelens P.G., van Norren K., van Leeuwen P.A. (2001). Flavonoids: A review of probable mechanisms of action and potential applications. Am. J. Clin. Nutr..

[23] He F., Mu L., Yan G.L., Liang N.N., Pan Q.H., Wang J., Reeves M.J., Duan C.Q. (2010). Biosynthesis of anthocyanins and their regulation in colored grapes. Molecules.

[24] Veitch N.C., Grayer R.J. (2008). Flavonoids and their glycosides, including anthocyanins. Nat. Prod. Rep..

[25] Yang M., Koo S.I., Song W.O., Chun O.K. (2011). Food matrix affecting anthocyanin bioavailability: Review. Curr. Med. Chem..

[26] Kim T.J., Lee K.B., Baek S.-A., Choi J., Ha S.-H., Lim S.-H., Park S.-Y., Yeo Y., Park S.U., Kim J.K. (2015). Determination of lipophilic metabolites for species discrimination and quality assessment of nine leafy vegetables. J. Korean Soc. Appl. Biol. Chem..

[27] Ruzzo E.K., Capo-Chichi J.-M., Ben-Zeev B., Chitayat D., Mao H., Pappas A.L., Hitomi Y., Lu Y.-F., Yao X., Hamdan F.F. (2013). Deficiency of asparagine synthetase causes congenital microcephaly and a progressive form of encephalopathy. Neuron.

[28] Kim Y.B., Park S.Y., Park C.H., Park W.T., Kim S.-J., Ha S.-H., Arasu M.V., Al-Dhabi N.A., Kim J.K., Park S.U. (2016). Metabolomics of differently colored Gladiolus cultivars. Appl. Biol. Chem..

[29] Kim J.K., Park S.-Y., Lee S.M., Lim S.-H., Kim H.J., Oh S.-D., Yeo Y., Cho H.S., Ha S.-H. (2013). Unintended polar metabolite profiling of carotenoid-biofortified transgenic rice reveals substantial equivalence to its non-transgenic counterpart. Plant Biotechnol. Rep..

[30] Jeon J., Kim N.Y., Kim S., Kang N.Y., Novak O., Ku S.J., Cho C., Lee D.J., Lee E.J., Strnad M. (2010). A subset of cytokinin two-component signaling system plays a role in cold temperature stress response in Arabidopsis. J. Biol. Chem..

[31] Wu X., Prior R.L. (2005). Identification and characterization of anthocyanins by high-performance liquid chromatography-electrospray ionization-tandem mass spectrometry in common foods in the United States: Vegetables, nuts and grains. J. Agric. Food. Chem..

[32] Charron C.S., Clevidence B.A., Britz S.J., Novotny J.A. (2007). Effect of dose size on bioavailability of acylated and nonacylated anthocyanins from red cabbage (*Brassica oleracea* L. Var. capitata). J. Agric. Food Chem..

[33] Kim M.S., Baek S.-H., Park S.U., Im K.-H., Kim J.K. (2017). Targeted metabolite profiling to evaluate unintended metabolic changes of genetic modification in resveratrol-enriched rice (*Oryza sativa* L.). Appl. Biol. Chem..

