# Supplementary material for: Comparative Metabolic Profiling of Green and Purple Pakchoi (Brassica Rapa Subsp. Chinensis)
Source: Molecules. 2018 Jul 2;23(7):1613. doi: 10.3390/molecules23071613 (PMC6099486; doi:10.3390/molecules23071613)
Supplement: Supplementary file 1 [file molecules-23-01613-s001.pdf]

Table S1. Chromatographic and spectrometric data of the metabolites identified by GC-TOFMS.

Compound	RT[1)]	RRT[2)]	Mass fragment[3)] (*m/z*)	Selected ion for quantification[4)] (*m/z*)
Lactic acid	4.476	0.426	117, **147**, 191	147
Alanine	5.176	0.492	**116**, 147, 190	116
Glycolic acid	6.238	0.593	**147**, 177, 205	147
Valine	6.308	0.600	**144**, 156, 218	144
Serine	6.573	0.625	**116**, 132, 147	116
Ethanolamine	7.022	0.668	100, 147, **174**	174
Glycerol	7.032	0.669	103, 117, **147**	147
Leucine	7.043	0.670	102, 147, **158**	158
Isoleucine	7.175	0.683	147, **158**, 218	158
Proline	7.226	0.687	**142**, 158, 216	142
Nicotinic acid	7.251	0.690	106, 136, **180**	180
Glycine	7.260	0.691	147, **174**, 248	174
Succinic acid	7.303	0.695	129, **147**, 247	147
Glyceric acid	7.363	0.701	133, **147**, 189	147
Fumaric acid	7.509	0.714	143, 147, **245**	245
Serine	7.536	0.717	147, **204**, 218	204
Threonine	8.077	0.768	101, 117, **219**	219
β-Alanine	8.332	0.793	147, **174**, 248	174
Malic acid	9.036	0.860	**147**, 233, 245	147
Aspartic acid	9.186	0.874	**100**, 147, 232	100
Methionine	9.217	0.877	128, 147, **176**	176
Pyroglutamic acid	9.245	0.879	147, **156**, 230	156
4-Aminobutyric acid	9.262	0.881	147, **174**, 304	174
Threonic acid	9.349	0.889	**147**, 205, 220	147
Glutamic acid	10.069	0.958	128, 156, **246**	246
Phenylalanine	10.148	0.965	100, 192, **218**	218
Xylose	10.196	0.970	**103**, 147, 217	103
Asparagine	10.311	0.981	**116**, 132, 231	116
Ribitol (Internal Standard)	10.511	1.000	103, 147, **217**	217
Glutamine	11.177	1.063	147, **156**, 245	156
Shikimic acid	11.265	1.072	147, **204**, 255	204
Citric acid	11.341	1.079	147, **273**, 347	273
Quinic acid	11.492	1.093	**147**, 255, 345	345
Fructose	11.546	1.098	**103**, 147, 217	103
Fructose	11.584	1.102	**103**, 147, 217	103
Galactose	12.028	1.144	**147**, 205, 319	147
Glucose	12.053	1.147	**147**, 160, 205	147
Mannose	12.141	1.155	**147**, 205, 319	147
Inositol	13.227	1.258	147, 217, **305**	305
Ferulic acid	13.297	1.265	308, 323, **338**	338
Tryptophan	14.137	1.345	**202**, 219, 348	202
Sinapic acid	14.233	1.354	**338**, 353, 368	338
Sucrose	16.201	1.541	147, **217**, 361	217
Maltose	16.501	1.570	**147**, 204, 361	147
Trehalose	16.523	1.572	147, **191**, 361	191
Raffinose	20.037	1.906	204, **217**, 361	217

[1)]Retention time (min).

[2)]Relative retention time (retention time of the analyte/retention time of ribitol).

[3)]List of the first three ions with the highest intensities. Ions in boldface indicate the most intense product ion.

[4)]Specific ion mass used for quantification.

Table S2. Contents of organic acids and other metabolites in green and purple pakchoi.

	Green	Purple
Nicotinic acid	0.0063±0.0004	0.0159±0.0010**
Lactic acid	0.2099±0.0119	0.3644±0.0494**
Quinic acid	0.0096±0.0004	0.0356±0.0005**
Glyceric acid	0.7284±0.0162	0.0989±0.0057**
Citric acid	2.9172±0.0244	3.9266±0.0462**
Fumaric acid	0.3836±0.0186	0.2443±0.0222**
Succinic acid	0.5845±0.0385	0.5831±0.0089
Malic acid	17.2694±0.4403	13.2529±0.1346**

Contents of organic acids and other metabolites were measured in 2-month-old green and purple pakchoi (μg g^{-1} dry weight). Each value represents the mean of three technical replicates and error bars are SDs. Asterisks indicate significant differences the purple pakchoi compared with the green pakchoi using Student's *t* test (*P <0.05; **P < 0.01).

Table S3. Primers used in this work.

Gene	Primer sequence (5' to 3')	Size (bp)
BrPAL1 QRT (F)	GTTGGAAATGGTGTGAAGGTGG	181
BrPAL1 QRT (R)	CTTATAAGCTCCTTCTGAAGTGC	
BrPAL2 QRT (F)	CCCTCAGATCGAAGTGATCC	199
BrPAL2 QRT (R)	AATTGAGCGAACATGAGCTTCC	
BrC4H QRT (F)	ATCCTGGTCAACGCCTGGTG	145
BrC4H QRT (R)	GTCCAACACCAAACGGCACA	
Br4CL1 QRT (F)	CCCAATCACCTCCCTCTCCAC	133
Br4CL1 QRT (R)	GCGACATGGACGTCGGAGTAA	
BrCHS QRT (F)	AGGAAACGCCACATGCACCT	114
BrCHS QRT (R)	AGGGACTTCGACCACCACGA	
BrCHI1 QRT (F)	CTTGAATCGATCATTGGAAAGAACG	91
BrCHI1 QRT (R)	CCTTGTCACTATTCATCAGCTGAG	
BrF3H QRT (F)	CAAGCCACACGAGACGATGG	110
BrF3H QRT (R)	TTGAACCTCCCGTTGCTCAGA	
BrF3'H QRT (F)	GCCGGAGAAGCTGAACATGG	117
BrF3'H QRT (R)	TAAGCCGACCCGAGTCCGTA	
BrFLS QRT (F)	TCCTTCCGCCGTCATTGTTC	141
BrFLS QRT (R)	TCACGGTGTGGCTCCAAGAA	
BrDFR QRT (F)	GGACAAAGTTCCGGGCAGTG	140
BrDFR QRT (R)	TCTGCTGTGCCGACATGTGA	
BrANS QRT (F)	ATTACCCGAAATGCCCTCAG	241
BrANS QRT (R)	TTCTCCTTATTCACCAACCCAC	
BrEF1α QRT (F)	ATACCAGGCTTGAGCATACCG	117
BrEF1α QRT (R)	GCCAAAGAGGCCATCAGACAA	

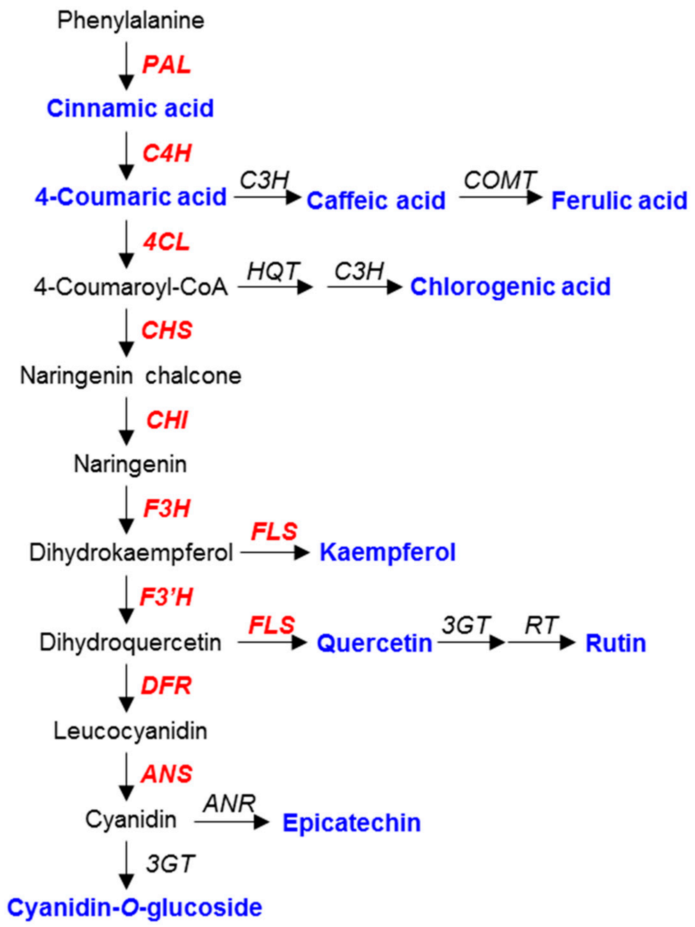

Figure S1. Schematic representation of the phenylpropanoid biosynthetic pathway in plants. PAL, phenylalanine ammonia-lyase; C4H, cinnamate 4-hydroxylase; 4CL, 4-coumarate-CoA ligase; CHS, chalcone synthase; CHI, chalcone isomerase; F3H, flavanone-3-hydroxylase; F3′H, flavonoid-3′-hydroxylase; FLS, flavonol synthase; DFR, dihydroflavonol reductase; ANS, anthocyanin synthase; 3GT, flavonoid 3-O-glucosyltransferase; RT, 3-O-rhamnosyltransferase; COMT, caffeic O-methyltransferase; HQT, hydroxycinnamoyl-CoA quinate hydroxycinnamoyltransferase; C3H, 4-coumarate 3-hyroxylase; and ANR, anthocyanidin reductase. Bold letters indicate the genes or compounds measured in this study.

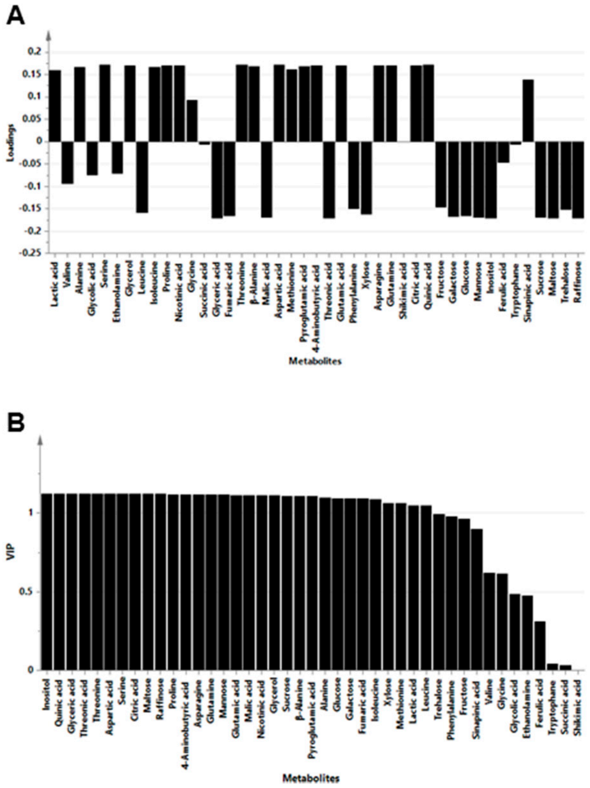

Figure S2. The loading plot (**A**), and influence variables used to create a discrimination model for green and purple pakchoi (**B**). Variable important in the projection (VIP) was identified from the OPLS-DA model.

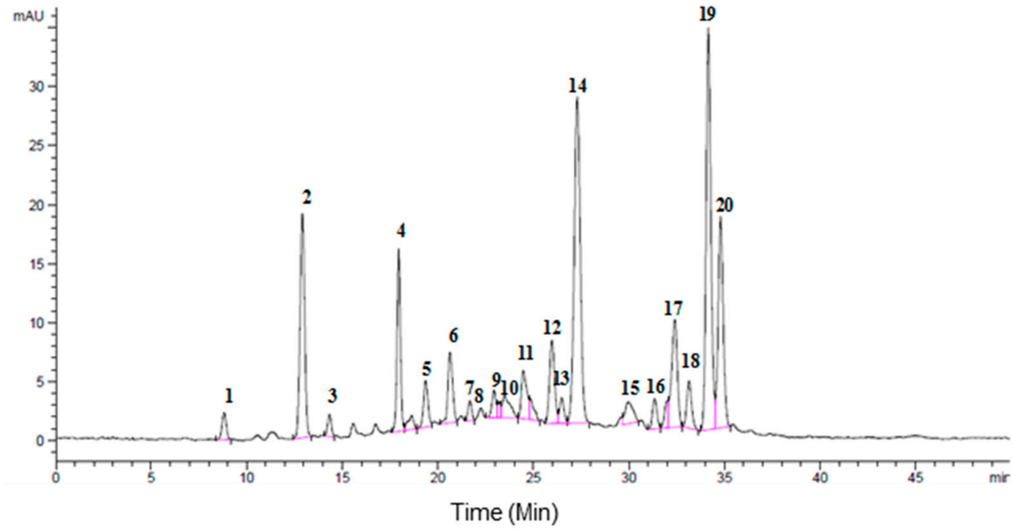

Figure S3. HPLC profiles of anthocyanins in the purple pakchoi (8389). The peak numbers indicate the anthocyanins in Table 2.
